# Supplementary material for: Quantitative Proteomic Analysis of Zearalenone-Induced Intestinal Damage in Weaned Piglets
Source: Toxins (Basel). 2022 Oct 13;14(10):702. doi: 10.3390/toxins14100702 (PMC9609629; doi:10.3390/toxins14100702)
Supplement: Supplementary file 1 [file toxins-14-00702-s001.zip › toxins-1953786 supplementary material-done.pdf]

toxins

MDPI

Article

Quantitative Proteomic Analysis of Zearalenone-Induced Intestinal Damage in Weaned Piglets

Lulu Ma, Yanping Jiang, Fuguang Lu, Shujing Wang, Mei Liu, Faxiao Liu, Libo Huang, Yang Li, Ning Jiao, Shuzhen Jiang, Xuejun Yuan, and Weiren Yang

Table S6. Ingredients and nutrient contents of the basal diet (air-dry basis)[1].

Ingredients	Content, %	Nutrients	Analyzed values, %
Expanded corn	64.43	Metabolizable energy，MJ/kg	13.86
Whey powder, CP 3%	5.0	Crude protein	18.48
Fermented soybean meal	14.0	Calcium	0.74
Expanded soybean	8.5	Total phosphorus	0.62
Fish meal, CP 63.28%	4.0	STTD phosphorus	0.41
$CaHPO_4$	1.15	ATTD phosphorus	0.38
Pulverized Limestone	0.7	Lysine	1.38
NaCl	0.2	Methionine	0.40
L-Lysine HCl	0.76	Sulfur amino acid	0.66
DL-Methionine	0.08	Threonine	0.85
L-Threonine	0.16	Tryptophan	0.23
L-Tryptophan	0.02		
Premix[1]	1.00		
Total	100.00		

[1]Supplied per kilogram of diet: vitamin A, 3300 IU; vitamin D_3, 330 IU; vitamin E, 24 IU; vitamin K_3, 0.75 mg; vitamin B_1, 1.50 mg; vitamin B_2, 5.25 mg; vitamin B_6, 2.25mg; vitamin B_{12}, 0.026 mg; pantothenic acid, 15.00 mg; niacin, 22.50 mg; biotin, 0.075 mg; folic acid, 0.45 mg; Mn ($MnSO_4 \cdot H_2O$), 4.00 mg; Fe ($FeSO_4 \cdot H_2O$), 90 mg; Zn ($ZnSO_4 \cdot H_2O$), 90 mg; Cu ($CuSO_4 \cdot 5H_2O$), 6.00 mg; I (KIO_3), 0.14 mg; Se (Na_2SeO_3), 0.30 mg. STTD, standardized total tract digestible; ATTD, apparent total tract digestible.

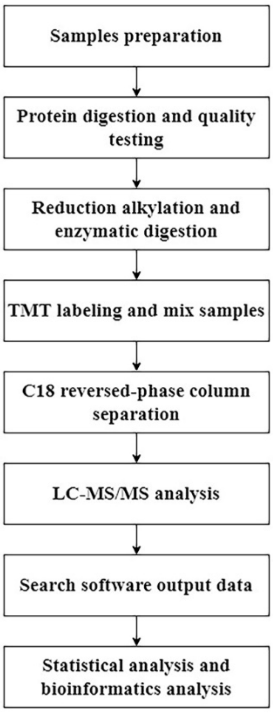

Figure S1. Screenshot of the workflow of Proteome Discoverer.
